# Breast cancer metastasis to thyroid: a retrospective analysis

**DOI:** 10.4314/ahs.v17i4.11

**Published:** 2017-12

**Authors:** Lingyan Zhou, Liyu Chen, Dong Xu, Qi Shao, Zhenying Guo, Minghua Ge

**Affiliations:** 1 Department of Ultrasound, Zhe Jiang Cancer Hospital affiliated to Zhejiang Chinese Medical University, Guangji road 38, GongShuQu, Hangzhou 310022, Zhejiang, China; 2 Department of Pathology, Zhe Jiang Cancer Hospital, Hangzhou, Zhe jiang province, China; 3 Department of Head and Neck surgery, Zhe Jiang Cancer Hospital, Hangzhou, Zhe jiang province, China

**Keywords:** Thyroid, ultrasonography, breast cancer, metastasis

## Abstract

**Background:**

Breast cancers metastasizing to thyroid gland are relatively uncommon in clinical practice.

**Objective:**

Retrospective analysis of data from breast cancer patients with thyroid metastasis (TM).

**Methods:**

The US suspected, fine-needle aspiration cytology (FNAC) confirmed TM in breast cancer patients, treated between 2005 and 2015 at our hospital, was retrospectively analyzed. The data were re-evaluated by the pathologist and radiologist who were blinded to the patients' data.

**Results:**

FNAC and immunohistochemistry confirmed the ultrasonography (US) suspected TM in eight breast cancer patients. Clinically both unilateral and bilateral TM was seen, which were symptomless and metachronously (6–121 months) metastasized. Six of eight cases exhibited recurrence/distant metastasis and were treated with chemotherapy/thyroidectomy of which two cases passed away. The remaining two patients had no recurrences/distant metastases and were treated with partial/total thyroidectomy. Post-chemotherapy US showed more homogenous thyroid parenchyma with gathering of calcification that reduced in size, revealing the sensitiveness of TM to chemotherapy.

**Conclusion:**

US was useful in screening TM in breast cancer patients. Both partial and total thyroidectomy was effective in disease free survival of isolated TM cases, with controlled primary condition. TM responded well to chemotherapy in most of the recurrent breast cancer cases with or without distant metastasis.

## Introduction

Breast cancer is the leading cause of cancer related death in women. In spite of its relatively early detection and treatment, distant metastasis to various tissues, including thyroid gland, still remains a challenge. In general, despite its high vascularization, metastasis to thyroid gland is uncommon (incidence of 0–5% in non-malignant cases to about 24% in malignant cases) and mostly metachronous, as reported in series of autopsy studies.^1^–^7^ Metachronous thyroid metastasis is defined as thyroid metastasis observed at or greater than 6 months after the diagnosis of primary cancer. Following the pattern, reports of breast cancer metastasizing to thyroid gland are relatively uncommon in clinical practice too; however, breast carcinoma is one of the common primary tumor of thyroid metastasis, seen at post-mortem.^8^ With this rarity, added with metachronous metastasis nature, particularly many years after the diagnosis of initial tumor, can pose a diagnostic challenge. Also, as treatment strategies used to treat primary and metastatic thyroid malignancies vary, it becomes essential to appropriately distinguish primary thyroid cancer from metastatic thyroid cancer. In this regard, routine US assessment of thyroid gland, followed by FNAC on suspicion, is part of the breast cancer patients' diagnostic regimen in our hospital through which TM patients are identified. The objective of present study was to analyze the ultrasonographic and clinical features of TM condition in breast cancer patients, through which we wish to contribute to the existing understanding of this rare condition.

## Materials and methods

The study was approved by our hospital ethics committee, reference number - IRB-2015-243, who waived the need for informed consent procedure. All the patients' personal identification information was removed before study related usage.

### Patients

Between 2005 and 2015, routine US analysis of breast cancer patients revealed seven cases with radiographic changes in their thyroid architecture, who underwent US-guided fine-needle aspiration cytology (FNAC) confirming TM. The US-guided FNAC was performed using a 5ml syringe, with 1∼2ml negative press, targeting the region that seemed to show the most compact area of sonographically visible microcalcifications or nodules, and thus suspicious for malignancy. An eighth case of TM, diagnosed and treated at a different hospital, visited our hospital for follow up, whose data we also considered in this analysis.

### Conventional US

The obtained conventional US reports of all patients were performed using the wide-band 5–10 MHz linear probe on a single ultrasound machine (Philips IU22, Philips Medical System, Bothell, WA, USA) by three different radiologists with over eight years of experience. The high-resolution images were documented/stored in PACS system.

For the study purpose, to reduce the inter-observer variability, all the images were retrospectively reviewed by a radiologist (>15 years of experience) who was blinded to the clinical, radiological and pathological findings. The US findings were assessed for: echogenicity of thyroid parenchyma, the appearance of the lesions (with mass or without mass), mass details (size, contour, margin, echogenicity, internal structure and vascularity) and presence of calcification. Any discrepancy was resolved through discussion with the respective radiologist who provided the prior diagnosis.

### Tissue processing and analysis

Smear slides, prepared from the thyroid gland aspirate, were subject to histological [hematoxylin and eosin (H&E) stain] and immunohistochemical [thyroglobulin (TG) and thyroid transcription factor — 1 (TTF-1)] analysis. FNAC was also performed for “recurrent foci at chest wall” which served as a reference standard for cellular morphological assessments. In addition to the above stains, the thyroid tissue samples obtained from thyroidectomy in 2 patients were subject to immunohistochemical analysis for estrogen receptor (ER) and Progesterone receptor (PR). The slides were read by two qualified and experienced (>25 years) pathologists providing their diagnosis. For the study purpose, the H&E and immunohistochemistry images of all the cases were re-read by a pathologist (>11 years of experience) who was blinded to the prior diagnosis and other patient data. Any discrepancy was resolved through discussion with the respective pathologist who provided the prior diagnosis. Clinical records of all the patients were further analyzed for details of the primary condition, interval between primary condition diagnosis and TM, serum thyroid hormone level, primary pathology, other associated metastasis, therapeutic strategy undertaken, and survival time after diagnosis.

## Results

### Clinical findings

The study comprised of 8 female patients who were diagnosed with TM from breast cancer between 2005 and 2015. The mean age at the diagnosis of TM was 55.37±9.33 years (range of 43 to 69 years). In all the subjects, the condition was symptomless at the time of diagnosis and was suspected of TM during routine US examination.

Clinically, all the subjects had a history of breast cancer and had received treatment for the same. Clinical details of the primary condition are detailed under table 1.

**Table 1:** Clinical details of the primary breast cancer condition

case	Breast cancer location	Type of breast cancer	Size of the breast tumors	IHC for breast cancer	associated metastasis during the diagnosis of breast cancer	Treatment given to breast cancer when it was diagnosed	prognosis with respect to breast cancer
1	Bilateral	poorly differentiated adenocarcinoma	L:42mm R:21mm	P53 (+), Her2 (3+), ER (-), PR(+,approximately 10%), Ki67 (+, 40%)	5 axillary lymph and left chest wall	four cycles (GH regimens: Gemcitabine 1.8g,d1; Herceptin 6mg/kg,d1), than Radiotherapy (DT 50GY/25F); Leuprorelin Acetate For Injiction 3.75mg and Arimidex 1mg qd.	Partial Response
2	Right	Infiltrating adenocarcinoma	15mm	Her2 (2+), ER (-), PR(-), Ki67 (+, 40%)	3 axillary lymph	sequential therapy*4times (Pirarubicin, 80mg, d1;CTX 1.0,d1), and Docetaxel 180mg d1;after that, radiotherapy was given, DT 50 GY/25FX/35D	Complete Response
3	Right	Signet ring cell carcinoma	31mm	P53 (-), Her2 (-), ER (+), PR(-), Ki67 (+)	21 axillary lymph	CTX 0.8 d1,5;5-FU,0.5 d1-3;TPH60mg; radiotherapy	Complete Response
4	Bilatera	infiltrating ductal carcinoma	L:17mm R:20mm	P53 (2+), Her2 (2+), ER (-), PR(-), Ki67 (+, 5%)	5 right axillary lymph and left chest wall	four cycles (CT regimens: CTX 0.6 d1, Docetaxel:80mg,d1);	Complete Response
5	Left	infiltrating ductal carcinoma	18mm	ER(-),PR(-),P53(3+),Her2(+)	6 axillary lymph	CTX0.6,Epi-ADM 90µg, 5-FU 0.75 d1	Complete Response
6	Right	Low grade ductal carcinoma	30mm	P53 (-), Her2 (3+), ER (we ak+, 10%), PR(+, 1%), Ki67 (+, 15%)	4 axillary lymph	TC regimens (docetaxel, 130mg, d1; cyclophosphamide 0.75g, d1)(refuse Herceptin for economic reason)	Complete Response
7	Right	poorly differentiated adenocarcinoma	24mm	do not have IHC result because the patient underwent mastectomy 10 years ago	16 axillary lymph	Tamoxifen peroral	Complete Response
8	Right	medullary carcinoma	20mm+15mm	P53 (3+), Her2 (2+), ER (-), PR(-), Ki67 (+, 25%)	14 axillary lymph /nipple /skin	two cycles (THC regimens: 240 mg, Carboplatin 700mg; Herceptin 8mg/m2), TAX allergy,so chang to	Partial Response

The location of breast cancer of these 8 cases was as follows: right (n=5), left (n=1) and bilateral (n=2). The median interval from the diagnosis of breast cancer to detection of TM was 76.5 months (range - 6 to 121 months), and thus was labelled as “metachronous metastasis” in all the cases. The details of recurrence/distant metastasis associated with TM, the timeline at which they were diagnosed in respect to TM and their treatment regimen are detailed in table 2.

**Table 2:** Ultrasound and clinical findings of thyroid metastasis cases

Serial No.	Age (years)	Breast cancer location	Months to metastasize (in relation to primary condition diagnosis)	Metastases elsewhere or recurrences	the timeline (breast recurrence in relation to thyroid metastasis diagnosis)	US images of thyroid	Treatment	Interval between chemotherapy and ultrasound change (months)	Survival time after diagnosis (months)
1	48	Bilateral	87	chest wall	thyroid metastasis was diagnosed 20 months later than recurrence founded	heterogeneous and diffuse calcifications without nodule	chemotherapy	8	14 Alive
2	59	Right	27	chest wall	thyroid metastasis was diagnosed 11 months later than recurrence founded	heterogeneous and diffuse calcifications without nodule	chemotherapy	5	5 Alive
3	57	Right	108	Supraclavicular nodes, lung	thyroid metastasis and recurrence was diagnosed at the same time	heterogeneous and diffuse calcifications without nodule	chemotherapy	15	21
4	67	Bilateral	71	Axilla nodes	thyroid metastasis and recurrence was diagnosed at the same time	heterogeneous and diffuse calcifications without nodule	chemotherapy	4(follow up time is not long enough and we can only see the thyroid parenchyma homogenous )	4 Alive
5	48	Left	121	Lung	lung metastasis was found after thyroidectomy	hypoechoic solid nodule	total thyroidectomy	NA	15
6	52	Right	6	Nil	No recurrence when thyroid metastasis was diagnosed	hypoechoic solid nodular and nodular goiter	Right lobectomy	NA	45 Alive
7	69	Right	57	Nil	No recurrence when thyroid metastasis was diagnosed	heterogeneous and diffuse calcifications without nodule	total thyroidectomy	NA	38 Alive
8	43	Right	82	Cervical and mediastinal lymph node	thyroid metastasis was diagnosed 3months after the recurrence	Typical Hyperthyroidism architecture with diffuse calcifications	chemotherapy	9	30 Alive

Briefly, two patients presented with recurrence of the primary condition on the chest wall and were treated with chemotherapy. Four patients exhibited associated metastasis to other organs/tissues, such as the lungs and lymph nodes (supraclavicular, axillary, cervical and mediastinal lymph nodes). Three of these patients received chemotherapy while one underwent thyroidectomy (at a different hospital) for suspected malignant nodule. The remaining two patients (25%) showed no signs of recurrence or distant metastasis and were treated with partial thyroidectomy (right lobectomy) for one case and total thyroidectomy for the other. One patient, who showed distant metastases to cervical and mediastinal lymph nodes, also presented with a history of hyperthyroidism and showed slightly elevated serum thyroxine (T4) level (182 nmol/ml).

### Patient survival

The survival time of patients was considered from the date of their TM diagnosis to the completion of this study or till the patient died , whichever happened earlier (Table 2). Two recurrence patients with foci on the chest wall received chemotherapy and were alive at 14 and 5 months, respectively, from the date of their TM diagnosis. Of the four patients who exhibited distant metastasis, 3 received chemotherapy of which one patient died at 21 months while the remaining two were alive at 4 and 30 months. One of these four distant metastasis patients underwent total thyroidectomy (at a different hospital) with no chemotherapy and died at 15 months post-TM diagnosis. The remaining two patients with isolated TM had post-thyroidectomy disease free survival at 45 months (right lobectomy) and 38 months (total thyroidectomy), respectively, of their TM diagnosis.

### Histology/immunohistochemistry

**Fig 1:**
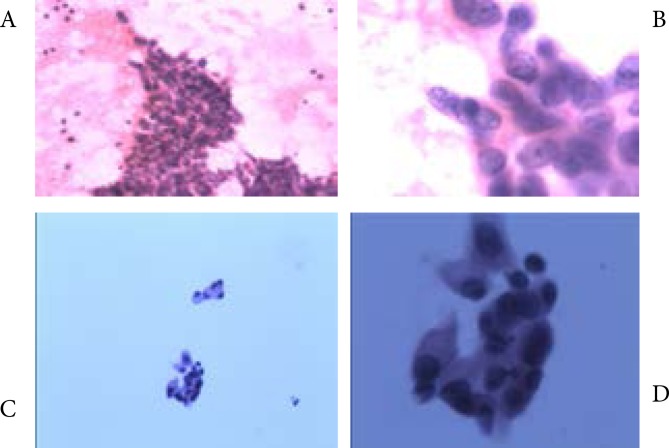
A&B. H&E stained FNAC smear samples of thyroid aspirate showing highly cellular smear with clusters of malignant epithelial cells, C&D. H&E stained FNAC smear samples of aspirate from “recurrent foci on chest wall” showing malignant epithelial cells.

The diagnosis from the study specific pathologist was in agreement with the patients' original histopathological diagnosis. Microscopic examination of H&E stained FNAC samples from thyroid aspirate revealed increased cellularity with clusters of malignant epithelial cells (Fig 1 A&B).

The cells were highly pleomorphic with increased Nucleus/Cytoplasm (N/C) ratio, vesicular nuclear chromatin with prominent nucleoli and abundant cytoplasm. The characteristic features of primary thyroid carcinoma, including papillary formation, nuclear grove and intranuclear pseudoinclusion, were absent. Also all the samples were found TG and TTF-1 negative revealing malignant cells of non-thyroid origin. The cellular morphology of all the FNAC samples matched to that of the malignant cells found in the aspirate from “recurrent foci on the chest wall” thus confirming the primary source of origin (Fig 1 C&D).

**Fig 2:**
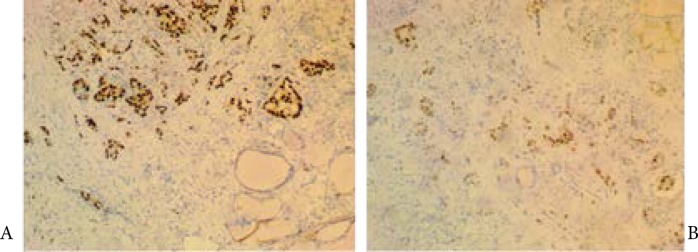
Immunohistochemistry of thyroid tissue samples, obtained during thyroidectomy, showing ER positive cells (A) in one sample and PR positive (B) in the other

Immunohistochemical analysis of thyroid tissue samples, obtained during thyroidectomy, showed ER positive cells in one sample and PR positive in the other, reconfirming the presence of malignant breast epithelial cells (Fig 2).

### US features of thyroid metastases in breast cancer patients

**Fig 3:**
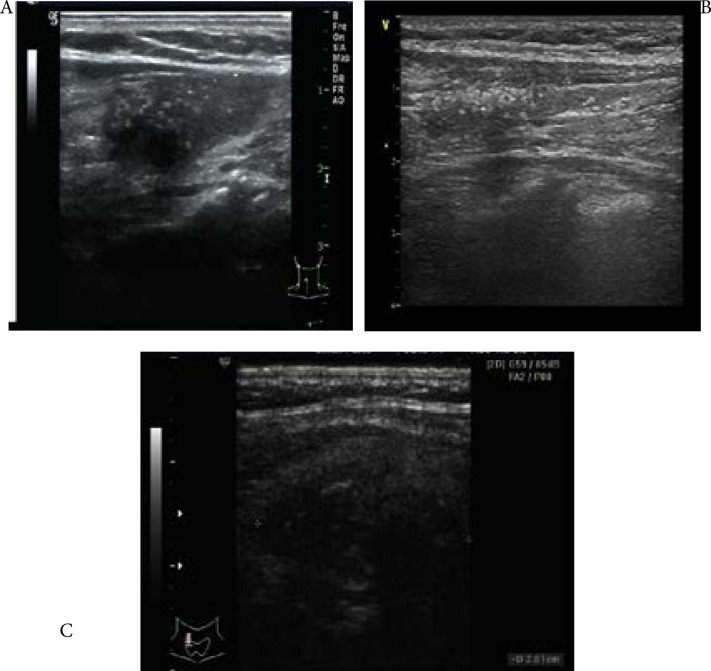
A. Heterogeneous appearance with diffuse calcifications of the thyroid parenchyma, B. reduction in the calcification with homogeneous appearance of thyroid parenchyma after chemotheraphy, C. hypoechoic solid node with irregular margins and multicalcifications

The readings from the study specific radiologist agreed with the patients' original radiological findings. US images of six of eight patients showed heterogeneous thyroid parenchyma with diffuse calcifications (Fig 3A). Five of these six patients showed no signs of nodules while one showed a hyperthyroidism associated nodule. Post-chemotherapy, the diffuse calcifications reduced and thyroid parenchyma appeared homogeneous in all the six patients (Fig 3B). The remaining two of eight patients showed hypoechoic solid node, with irregular margins and multicalcifications (Fig 3C), and one of them was comorbid with nodular goiter. US and clinical findings of all the eight patients are listed in table 2.

## Discussion

The thyroid gland has an affluent blood supply of about 560 mL/100 g tissue/min, which is second only to the adrenal gland. Yet, thyroid metastasis from the cancer of extra-thyroid origin is infrequent,^9^ and the reason for this is not clear. Chung et al., found that the abnormal thyroid conditions like goiter increases the probability of TM, which may be due to alteration in local homeostasis resulting in decreased oxygen and iodine content.^7^,^10^,^11^ In the present study, 2/8 cases showed such associated thyroid pathology; goiter and hyperthyroidism.

TM is usually observed in elderly individuals in their sixth and seventh decades of life.^12^,^13^ Data from the present study revealed a mean age of 55.4 years which is slightly younger than that presented in the literature. As per autopsy reports, the incidence of TM in patients who die as a result of malignancy is up to 24%.^14^ The prevalence of thyroid nodules ranges from 20–67%, and the incidence of malignant nodules is about 0.45–13%.^15^ In recent years the reports of TM cases has been gradually increasing, which may be related to more frequent thyroid imaging and FNAC studies in cancer patients.^16^–^18^

Most common sites of primary tumors are renal cell carcinoma, breast cancer and lung cancer, however, there is no complete agreement as to which cancer most frequently metastasize to the thyroid as it may depend on many factors such as epidemiology and clinical behavior of the primary cancer and diagnostic methods used.^8^ TM from renal cell carcinoma is usually symptomatic, where the patients present with symptoms such as a new neck mass, dysphagia and hoarseness, while that from lung and breast cancer may go symptomless.^8^ A study described an unusual case of thyroid metastasis from breast carcinoma, characterized by massive intra-arterial embolization and clinically presented as acute thyroiditis, which is uncommon. 19 Also TM can present as a synchronous ormetachronous manifestation of known primary tumors or a first finding of unknown primary tumor (occult primary neoplasm).^20^–^23^ All TM patients in our study were symptomless, demonstrated metachronous metastasis, and were discovered during routine US imaging procedures. Several studies have assessed the usefulness of US in predicting thyroid cancer, while characterizing its features, and recommend US evaluation as a good modality for early detection of thyroid cancer.^24^–^27^ Given the cost effectiveness of this non-invasive diagnostic tool, the inclusion of US analysis in routine follow-up of breast cancer patients could be of use in early detection of thyroid metastasis.

Ultrasound plays an important role in screening thyroid disease and making a differential diagnosis of benign and malignant tumors. US features of malignant thyroid nodules in general and of primary thyroid cancer and thyroid metastasis in particular are detailed elsewhere.^28^,^29^ Although no single mentioned feature is decisive for malignant thyroid condition, presence of a combination of two or more of these features increases the chances of malignancy. In our study, two TM samples appeared as classical Primary Thyroid Cancer (PTC), while six patients showed heterogenous thyroid parenchyma with diffuse calcifications without nodule, which is rare in TM conditions. Hence it becomes important to consider the possibility of metastasis from elsewhere while diagnosing new thyroid masses in patients with a previous history of malignancy. But it is difficult to differentiate between PTC and TM using US only. US guided FNAB/FNAC is of value in such conditions whose accuracy for TM diagnosis from breast cancer is reportedly 90.8% to 91.2%.^30^ In this regard, if malignant cells are present in a thyroid FNAB and primary malignancy is not a consideration, then clinical history consideration with immunohistochemical analysis is essential for accurate diagnosis.

The interval from a non-thyroid primary cancer diagnosis to TM diagnosis varies from a few months in aggressive malignancies, to many years in less aggressive condition, the median of which was reported as 53 months.^7^ The same was found to be 76.5 months (median interval) in our study which is considerably higher than the reported value. Additional findings from our study reveal that the location of primary source, including bilateral breast cancer condition, has no association with the probability of occurrence of TM.

The metachronosity of thyroid metastasis from breast cancer can be as long as 12 years from the diagnosis of the primary condition.^31^ In the present study, one of our patients presented to the hospital with TM, more than 10 years after she was diagnosed and treated for breast cancer. Such durations can be long enough to miss the past diagnosis/treatment of malignant disease thus demanding extra attention in the history.

Numerous case reports have suggested that metastases to the thyroid gland are associated with poor prognosis, 6,32 while others report that it does not seem to worsen the outcome when compared to other associated distant metastasis conditions.^33^ Few studies have assessed the effectiveness of the therapy for TM from breast cancer condition. In agreement with Ishikawa et al., we found that thyroidectomy in an isolated TM condition, with controlled primary tumor, may result in prolonged disease-free survival with no difference in survival time amongst total and partial thyroidectomy.^16^ But, total thyroidectomy was not effective in prolonging the life of a patient with lung metastasis. Chemotherapy is the treatment of choice in such patients with widespread distant metastatic condition. In the present study chemotherapy demonstrated shrinkage in the calcifications with a change in thyroid parenchyma from being heterogeneous to homogenous in four of five distant metastasis patients. Hence, our supposition is that for the controlled primary tumor condition, with no associated relapse or distant metastasis, thyroidectomy may form the treatment of choice in terms of disease free survival; while for the widespread metastatic condition the TM may respond well to the administered chemotherapy, thus eliminating the need for thyroidectomy.

Due to the rarity of the condition, the present study is limited by the number of patients assessed, which is not large enough to represent the population of breast cancer patients with TM. A long term follow-up is also desirable to assess the treatment (thyroidectomy/chemotherapy) value in long-term disease free survival. Such studies are further warranted.

## Conclusion

The infrequent, possibly metachronous and asymptomatic nature of TM from extra-thyroid origin, makes it a challenging condition to be diagnosed in a clinical setting. With breast cancer being one of the most common primary tumor exhibiting TM, a routine examination of the thyroid gland is essential in such patients. In this regards, the US analysis may serve as the screening tool of choice, owing to its non-invasive nature and cost effectiveness. None the less, it is difficult to differentiate between PTC and TM using US alone, during which US guided FNAB/FNAC is of value in obtaining a more accurate diagnosis. Thyroidectomy, both partial and total, could be the treatment of choice in a controlled primary tumor condition, with no associated relapse or distant metastasis; while in a widespread metastatic condition the TM may respond well to the chemotherapy, and thus may eliminate the need for thyroidectomy.
